# An Ecologically Valid Experimental Model for Repeated Evoked Pain Using a Wearable Device in Pain‐Free Individuals: A Pilot Feasibility Study

**DOI:** 10.1155/prm/9255164

**Published:** 2026-09-22

**Authors:** Merethe Blandhol, Adrian Nicolaisen Tiseth, Dan-Mikael Ellingsen

**Affiliations:** ^1^ Faculty of Medicine, University of Oslo, Oslo, Norway, uio.no; ^2^ Department of Physics and Computational Radiology, Division of Radiology and Nuclear Medicine, Oslo University Hospital, Oslo, Norway, oslo-universitetssykehus.no; ^3^ School of Health Sciences, Kristiania University of Applied Sciences, Oslo, Norway

## Abstract

Evoked experimental pain is a central method for studying basic pain mechanisms in humans. However, traditional methods of evoked pain delivered in controlled lab settings lack ecological validity and limits testing to short intervals. This study tested the feasibility, tolerability and construct validity of a new model for experimental evoked pain in participants’ daily environments, using a wearable device that delivered electric shocks to the wrist. Ten pain‐free individuals received 7 electric shocks over a period of one day (between 10:00 and 21:00) and completed ecological momentary assessment (EMA) following each stimulus. Shocks were individually calibrated to 4 out of 10 (NRS; 0 = *No shock felt*, 10 = *Shock is intolerable*). All participants completed the full study protocol, and no adverse events were reported. Overall, 84.1% of shocks were rated above 1.5 on pain intensity (VAS 0–10; 0 = *No pain*, 10 = *Extreme pain*). On average, pain intensity was rated 4.10 (SD = 1.99; VAS 0–10), consistent with individual calibration. Shock interference, rated retrospectively at the end‐of‐day EMA, was relatively low (*M* = 2.05, SD = 1.52; 0–10 VAS; range = 0–3.7), and most reported carrying out activities as planned. These results indicate that this model of experimental pain is feasible and tolerable for participants and successfully evokes pain in an ecologically valid setting. In future studies, this method may be applied over multiple days to investigate pain perception over time, with manipulating stimulus parameters, or in connection with daily activities.

## 1. Introduction

Experimentally evoked pain has long been a central method for studying the mechanisms of pain processing under controlled conditions. It has been extensively used to investigate mechanisms of somatosensory processing, including acute pain perception [1–3], the modulation of pain [4–7] and analgesic effects [8–12]. Experimentally induced noxious stimuli with controlled parameters (i.e., onset, stimulus intensity, body location) allow for the isolation of specific factors, thus strengthening internal validity. Standardised methods of pain administration, such as quantitative sensory testing (QST), have also been used to investigate group differences in pain perception [13], including sex differences [14–16], altered pain perception in clinical populations [17–20] and classification of clinical subgroups [21, 22].

Yet, current experimental models have several drawbacks that limit external validity, e.g., generalisability to real‐life acute or clinical pain. For one, the controlled lab environment limits the physical conditions under which pain is experienced. Participants often sit or lie stationary during stimulation, as most pain administration involves equipment that limits mobility. This differs greatly from most real‐life contexts where people experience pain, such as during movement or whilst performing daily activities. Moreover, the lab setting is an artificial context which emphasises study instructions, rating scales and experimental apparatuses—likely rendering the meaning of pain quite different from real‐life experiences. Furthermore, pain onset is often fairly predictable during lab‐based experiments, as the pain testing session has a limited duration. Even if the precise pain stimulation onset time is jittered or randomised, the participant still has a general expectation that pain will occur within a relatively short time. Yet, naturally occurring pain often happens without warning and is substantially more uncertain [23]. Lab‐based models also impose practical restrictions on how long participants can be present in the lab and how often they can return for repeated experiments. This makes it difficult to investigate temporal dynamics of pain, which are key to investigating mechanisms that unfold over time—like the transition from acute to chronic pain. Observational studies have *measured* these fluctuations in patients with chronic pain using momentary ratings in daily life [24, 25]. However, no current method exists for reliably *administering* or *manipulating* pain in naturalistic environments. Combining ecologically valid assessment and administration of pain would integrate the strength of experimental manipulation with the ecological validity of ambulatory assessments.

Here, we test the feasibility of a new ecologically valid model of experimentally evoked pain in ‘the wild’, which involves a wearable device that administers pain stimuli to participants in their daily environments. If feasible, this method may enable more accurate assessments of the influence of movement, activities, and temporal fluctuations. Moreover, this model may allow for longer experimental durations, over days or weeks, that can help uncover how pain perception changes as pain reoccurs. This enables, for instance, the exploration of the influence of mood or daily activities on pain perception and whether parameters of the pain stimuli (i.e., duration, intensity) influence pain perception differently depending on the time of day. To test whether such remote experimental pain administration is tolerable and feasible, we used a wearable device that gave mild‐to‐moderate electric shocks in a small sample of healthy volunteers over a period of one day. Variables including pain intensity, stimulus interference and mood were recorded using concurrent ecological momentary assessment (EMA), to evaluate tolerability, feasibility and construct validity and to explore possible reciprocal influences between pain and related factors. The aims of this study were therefore to test whether remote administration of repeated evoked electrical pain (1) is tolerable and feasible in a group of healthy volunteers and (2) has adequate construct validity in terms of reliably evoking pain. These research questions were evaluated using preregistered criteria.

## 2. Methods

### 2.1. Study Design

This pilot study employed a single‐arm noncomparative design, where all participants were assigned to the experimental condition (i.e., evoked pain administration). References to ‘randomisation’ describe counterbalancing protocols and not comparative group allocation. We applied a quasiexperimental basic research approach using experimentally evoked pain and measures of pain, mood and related outcomes, in order to investigate the pain modulatory system in healthy volunteers. Thus, the study was not considered to be a clinical trial testing a health‐related intervention, according to the ICMJE definition [26].

### 2.2. Evaluation Criteria

We established three criteria evaluating tolerability, feasibility and construct validity (preregistered DOI: 10.17605/OSF.IO/HPY2N), which served as go/no‐go criteria for further studies using this method: 1) For the model to have sufficient tolerability, the total participant completion rate should be at least 70%, after excluding withdrawals unrelated to the study procedure, and 2) there should be no serious adverse events related to the pain stimulus; 3) for the model to have sufficient feasibility and construct validity, i.e., reliably invokes pain, an average of at least 75% of stimuli (calculated by taking the percentage of all individual shock ratings across participants) should be rated above 1.5 on a pain intensity digital VAS scale (coded as 0 = *No pain*, 10 = *Extreme pain*; recorded to four decimals). Passing these 3 criteria would support the assumption that our remote electrical stimulation model is tolerable, and sufficiently and reliably evokes pain.

### 2.3. Sample

Healthy volunteers aged 18–65, without ongoing acute or chronic pain, a diagnosis of a serious health condition or ongoing mental illness, were eligible for the study. Participants were excluded if pregnant, unable to wear the watch or using medical equipment where mild electrical stimulation could interfere with functioning. Access to a smartphone was required, and all participants needed to agree to abstain from using heavy machinery, motorised vehicles, weapons or similar equipment during the study, where disruption from the electric shocks could pose a safety risk. The planned sample size was limited to 10 participants for pragmatic reasons, including limited resources and time. Participants were recruited using poster advertisements at local university buildings and through convenience sampling between March and July 2025. In total, 12 interested participants completed the online screening form and were eligible for participation. Two eligible participants did not respond when contacted, and a total of 10 participants were enrolled in the study (see Table 1 for sample characteristics).

**TABLE 1:** Descriptive statistics of gender, age and employment status of the sample along with baseline scores of standardised questionnaires.

		Mean (SD) or %	95% confidence interval	Score range	Range of scale
Demographics	Gender	70% female			
	Age	30.7 (5.89)	[26.5–34.9]	23–39	
	Employment	Full time (50%) Student (30%) Part time (20%)			

Baseline Questionnaires	Pain Catastrophising Scale (PCS)	10.6 (8.9)	[4.2–17.0]	0–25	0–52
	Anxiety (GAD‐7)	3.9 (2.1)	[2.4–5.4]	1–7	0–21
	Depression (BDI‐SF)	0.7 (0.9)	[0.02–1.4]	0–3	0–21
	Optimism (LOT‐R)	18.2 (2.9)	[16.1–20.3]	15–24	0–24
	Perceived Stress Scale (PSS)	4.3 (2.2)	[2.7–5.9]	1–8	0–16
	Life Satisfaction (LiSat‐11)	49.8 (5.7)	[45.8–53.8]	40–57	9–66

*Note:* LOT‐R, Revised Life Orientation Test; LiSat‐11, Life‐Satisfaction Questionnaire.

Abbreviations: BDI‐SF, Beck’s Depression Inventory Short‐Form; GAD‐7, Generalised Anxiety Disorder‐7; PCS, Pain Catastrophising Scale; PSS, Perceived Stress Scale.

### 2.4. Procedure

Participants who filled in an online screening form and were deemed eligible were invited for an in‐person study visit. At the first visit, participants signed informed consent, and the wearable device and necessary apps were set up before completing an individual pain calibration procedure and questionnaire measures. The participants wore the device for one day between the hours of 10:00 and 21:00, during which they received a total of 7 shocks in a pseudorandomised order and completed EMA following each shock. Participants were allocated to one of two protocols (A or B; see Supporting Table 1 for exact timepoints) with different time schedules to counterbalance any potential order effects and were blinded to the timings of each alarm. Random allocation was assigned by the researcher ahead of data collection by using computer‐based random shuffling of 10 A or B allocations (1:1 ratio; *N* = 5 participants in each group) paired to planned (*N* = 10) participant IDs. At the study visit, the researcher would schedule alarms based on this prior allocation, following Protocol A or Protocol B timepoints accordingly. After wearing the device for a full day, participants completed a second in‐person study visit including a debrief interview and a questionnaire about their experience. Participants were instructed to report any adverse events (i.e., negative events related to the study procedure), such as rashes or skin irritation from wearing the watch, intolerable pain or psychological distress, to researchers if they occurred. It was also made clear that the watch could be removed at any point, if necessary. No modifications to the protocol occurred during the study.

### 2.5. Remote Pain Administration

#### 2.5.1. Wearable Device

For evoking pain, we used the Pavlok 3 Pro [27], a commercially available watch that delivers mild electric shocks. The device is worn on the wrist and connects to a smartphone application via Bluetooth to allow for adjustment of shock strength, number of consecutive shocks and shock onset through scheduling ‘alarms’ to trigger either a shock, audible tone, vibration or a combination of the three. Electric shocks have an adjustable intensity (scaled from 5% to 100%; 136V, ∼0 mA minimum and 610V, ∼5.6 mA maximum) that can be adjusted in increments of 5%. After individual pain calibration (see below), future ‘alarms’ were scheduled in the app by the researcher according to the preset timepoints (Protocol A or B) using the Pavlok app installed on the participant’s phone. ‘Alarms’ then triggered one electric shock to the watch worn by the participant at the scheduled time. Due to limitations in the Pavlok app, it was not possible to objectively confirm the successful delivery or precise timing of the administration of individual shocks. Thus, self‐reported detection ratings by the participants were used as subjective confirmation. Participants turned off ‘alarms’ manually using a button press on the watch hardware and were instructed not to look at scheduled ‘alarms’ in the app. Shocks that were not disabled with a manual button press were repeated after 120 s until silenced. Currently, no independent validation of the device output or accuracy is publicly available.

#### 2.5.2. Individual Pain Calibration

Each participant went through a brief calibration procedure to ensure shock intensity was approximately 4 out of 10 on a verbal numeric rating scale (NRS) (0 = *No shock felt*, 10 = *Shock is intolerable* and participant wants to remove watch immediately). First, the watch was properly fitted on their nondominant wrist to ensure both adequate electrode contact and comfort. Participants were instructed to use the same notch on the wristband throughout the study. Before calibration began, participants were informed that they would receive multiple verbally announced shocks, with a short break between each, and rate each using the 0–10 pain intensity NRS.

Electric stimulation began at around 10%–20% intensity and ramped up in increments of 5%–10% until reaching an NRS = 8, after which the intensity was decreased until shocks were consistently rated close to an NRS = 4. The watch was then switched to the opposite wrist to control for potential sensitisation or habituation, and calibration continued until the selected intensity yielded a stable rating of around 4. The watch was then moved back to their nondominant arm and the procedure continued, adjusting the previous intensity, if necessary, until consistent ratings (NRS = 4) were given for 2‐3 consecutive stimulations.

Finally, participants were informed that a couple of unannounced shocks would be given. One unannounced shock calibrated at NRS = 4 was delivered whilst the participant was distracted. If necessary, the shock was repeated and adjusted to confirm consistent NRS = 4 intensity.

### 2.6. EMA

EMA questionnaires were administered through the m‐Path smartphone application [28, 29] following each shock. Participants were first asked whether they had felt a shock (‘Did you receive a shock?’, Yes/No). If they answered ‘yes’, they were asked about its pain intensity (‘How painful was the shock?’, VAS 0 = *No pain*, 10 = *Extreme pain*), unpleasantness (‘How unpleasant was the shock?’, VAS 0 = *Not at all*, 10 = *Extremely unpleasant*) and predictability (‘How surprising was it to receive a shock?’, VAS 0 = *Not at all*, 10 = *Extremely surprising*). For those reporting not having felt a shock, a question about possible reasons was asked (‘Were any of these the case when you didn’t receive a shock?’ Multiple choice: 1 = I didn’t wear the watch, 2 = The watch had no battery, 3 = I wore the watch, but didn’t feel anything, 4 = The watch was not connected to the phone, 5 = Other reason). Next, participants were asked about their current mood (‘To what extent do you feel: [happy/relaxed/sad/irritable/nervous]?’, VAS 0 = *Not at all*, 10 = *To a large extent*). During the last questionnaire of the day, participants were additionally asked how often they thought about the watch or receiving a shock throughout the day (‘How often did you think about the watch or that you would be shocked throughout the day?’, VAS 0 = *Not at all*, 10 = *To a large extent*) and to what extent the shocks interfered with their activities throughout the day (‘How much did the shocks impact your activities or tasks throughout the day?’, VAS 0 = *Not at all*, 10 = *Extremely much*). A questionnaire notification was sent 1 min after each shock delivery, and a reminder was sent after 5 min. Participants had 30 min to complete each questionnaire.

### 2.7. Questionnaires

#### 2.7.1. Baseline

At baseline, participants completed validated questionnaires on pain catastrophising (Pain Catastrophising Scale [PCS]) [30], anxiety (Generalised Anxiety Disorder [GAD‐7]) [31], depression (Beck’s Depression Inventory Short‐Form [BDI‐SF]) [32], optimism (Revised Life Orientation Test [LOT‐R]) [33], perceived stress (Perceived Stress Scale [PSS]) [34] and life satisfaction (Life‐Satisfaction Questionnaire [LiSat‐11]) [35].

#### 2.7.2. Postintervention

At the last visit, participants completed customised questions about their experience wearing the watch. These questions included ratings of average pain intensity of the shocks (‘On average, how painful were the shocks you received throughout the day?’, VAS 0 = *No pain*, 10 = *Extreme pain*), whether the shocks were too mild or too strong (‘On average the shocks were…’, Multiple choice: 1 = *Far too mild*, 2 = *Slightly too mild*, 3 = *Just right*, 4 = *Slightly too strong*, 5 = *Far too strong*), whether shocks were predictable (‘How often did you foresee you would get shocked?’, Multiple choice: 1 = *Never*, 2 = *Sometimes*, 3 = *Often*, 4 = *Almost every time*), whether participants checked upcoming alarms in the app (‘How often did you check the app to see when the next shock would be?’, Multiple choice: 1 = *Never*, 2 = *Sometimes*, 3 = *Often*, 4 = *All the time*), how disruptive the shocks were to planned daily activities (‘On average, how disruptive were the shocks for your activities and plans?’, VAS 0 = *Not at all*, 10 = *Extremely disruptive*) and how many activities or plans they avoided during participation (‘How many activities or plans did you avoid because of the shocks?’, Multiple choice: 1 = *None*, 2 = *Some*, 3 = *Many*, 4 = *Almost all*).

### 2.8. Debrief Interviews

Additionally, short debrief interviews were conducted at the last visit, asking participants about the disruptiveness of the shocks, any changes to daily routines, how participants would describe the sensation of the shocks, and whether they would be willing to wear the watch for longer.

### 2.9. Statistical Analyses

Analysis of data was conducted using RStudio (v. 2026.01.0) [36]. Visualisations of data were created using the ggplot2 R package [37]. EMA data collected using VAS scales were rescaled during analysis to a 0–10 scale with four decimal places (rounded to two decimals for simpler interpretation). For summary statistics of stimulus‐related outcomes (i.e., pain intensity, shock unpleasantness, shock surprise), the statistic was calculated using the total number of shocks felt (i.e., missed shocks not included), whereas summary statistics for mood variables used the number of completed EMA responses.

### 2.10. Approvals

The project was reviewed by the Regional Committee for Medical and Health Research (REK #739449) and was deemed outside the mandate and therefore exempt from approval. The study protocol and data protection protocol were approved by the data protection officer at the Oslo University Hospital (#24/11500), and the study was conducted in accordance with the Declaration of Helsinki.

## 3. Results

### 3.1. Demographics and Sample Characteristics

A total of ten participants were enrolled in the study, a majority of whom were female (70%). The age of participants ranged from 23 to 39 years (*M* = 30.7, SD = 5.89, 95% CI [26.5–34.9]), and 50% had full‐time employment at the time of participation. Baseline scores on standardised questionnaires showed generally low scores for pain catastrophising, anxiety, depression and perceived stress, whereas scores for optimism and life satisfaction were generally high. A descriptive summary of demographic information and baseline questionnaire scores is found in Table 1. Potential differences between time protocols (A and B) were visually inspected (see Supporting Figure 1). Due to the small sample size, data from both protocols were analysed together.

### 3.2. Completion Rate, Adherence and Adverse Events (Criteria 1 and 2)

All ten participants who were enrolled in the study completed all protocols, resulting in a protocol completion rate of 100%. All EMA questionnaires were answered (100% response rate), though some shock stimuli were missed for 3 of the participants (see 3.3.1 below). No adverse events were reported to the researchers throughout the study. The preregistered go/no‐go criteria relating to tolerability of the study protocol were therefore both fulfilled. Furthermore, participants were asked postintervention to disclose how often they checked the app to see when upcoming shocks were scheduled, to check blinding of shock onsets. All participants (*N* = 10) answered that they never checked the app to see the alarms, which suggests overall good adherence.

### 3.3. Quality Control/Construct Validity (Criterion 3)

#### 3.3.1. Shock Calibration and Detection

During calibration, the strength of shocks that corresponded to an NRS = 4 rating ranged from 25% to 90% (of 5%–100% possible; *M* = 51.5, SD = 20.15, 95% CI [37.1–65.9]) across participants (see Table 2 for an overview of individual shock calibration levels and ratings). Overall, 64 (91.4%) of 70 total administered shocks were detected by participants, with three participants missing 1–3 shocks each during participation. Reports of missed shocks most often stated that the watch was worn but no shock was felt (83.3% of missed shocks), whilst one participant reported that the watch was disconnected at the time.

**TABLE 2:** Individual overview of stimulus‐related variables.

	P1	P2	P3	P4	P5	P6	P7	P8	P9	P10	*M* (SD)/mode	95% CI	Range
Watch shock intensity setting (5%–100%)	60%	60%	30%	40%	75%	90%	25%	45%	50%	40%	51.5 (20.1)	37.1–65.9	25–90
Percentage of shocks felt (%)	100%	100%	71%	100%	100%	100%	57%	100%	100%	86%	91.4 (15.4)	80.4–102.4	57–100
Average pain intensity (VAS 0–10)	3.28	5.27	5.87	4.14	4.22	1.90	2.50	6.40	2.03	5.41	4.10 (1.99)	3.61–4.60	1.90–6.40
Average shock unpleasantness (VAS 0–10)	4.06	6.21	6.16	4.32	4.42	1.77	2.60	6.98	3.73	5.71	4.62 (2.10)	4.10–5.15	1.77–6.98
Average surprise of shocks (VAS 0–10)	8.20	7.89	6.63	7.39	5.30	4.45	4.25	8.90	6.54	6.55	6.72 (1.99)	6.22–7.22	4.25–8.90
Shock pain intensity rated above VAS 1.5 (%)	100%	100%	71%	86%	100%	57%	57%	100%	71%	86%	82.8 (17.7)	70.2–95.4	57–100
Attention to watch/shocks (VAS 0–10)	2.81	6.24	6.24	2.62	4.40	1.56	7.57	0.00	3.37	6.94	4.24 (2.59)	2.39–6.09	0–7.57
Shock interference (VAS 0–10)	3.66	3.39	3.52	2.06	3.32	0.00	3.02	0.85	0.00	0.65	2.05 (1.52)	0.96–3.14	0–3.66
Retrospective shock strength (multiple‐choice)	Just right	Just right	Just right	Slightly too mild	Just right	Slightly too mild	Slightly too mild	Slightly too strong	Slightly too mild	Just right	Just right (50%)		

*Note:* The watch shock intensity setting represents the strength of shock set in the app that corresponded to a participant rating of NRS = 4 during calibration. The table also shows the percentage of shocks felt during participation, the average (across shocks) reported pain intensity, shock unpleasantness and surprise of shocks. The percentage of shocks that were rated above 1.5 on a 0–10 pain intensity VAS for each individual is shown (out of 7 total shocks), which differs slightly from the pooled percentage calculation used for the preregistered quality control criterion (84.1% of all stimuli across individuals were rated > 1.5). How much attention was given to the watch and/or shocks during the day, and the level of shock interference with daily activities were both assessed once per day at the last questionnaire of the day (after the last shock). Retrospective recall of whether shocks were on average too mild or too strong was rated at the last study visit through an online questionnaire. Range refers to the range of individual means.

Abbreviation: CI, confidence intervals.

#### 3.3.2. Pain Intensity and Unpleasantness Ratings

When evaluating the preregistered quality control criterion that 75% of stimuli should be rated above a 1.5 on a 0–10 VAS pain intensity scale (calculated as the percentage of all shocks across participants), we opted to include shocks that were missed even though the watch was worn at the time in the denominator (their pain intensity counted as 0), in case these were missed because the calibrated intensity was too low. After excluding one missed shock from the denominator (due to disconnection), 84.1% of shocks (*N* = 58 of 69 total) were rated > 1.5, thus satisfying the quality control criterion. The average pain intensity of shocks across all participants, as measured using EMA, was 4.10 (SD = 1.99; VAS 0–10, 95% CI [3.61–4.60]), and average unpleasantness was 4.62 (SD = 2.10; VAS 0–10, 95% CI [4.10–5.15]).

The postintervention questionnaire asked participants to retrospectively rate the average pain intensity of shocks on a 0–10 VAS (*M = *3.9, SD = 1.65, range = 0.9–6.0, 95% CI [2.74–5.10]), showing similar ratings to the EMA. Participants also rated whether shocks were on average too strong or too mild using a 5‐option single‐select multiple‐choice question. Here, 50% of participants answered that shocks were ‘just right’ in strength, whereas 10% reported they were ‘slightly too strong’ and 40% reported they were ‘slightly too mild’.

#### 3.3.3. Shock Predictability and Surprise

On average, participants reported moderately high levels of surprise when receiving a shock (*M* = 6.72, SD = 1.99, 95% CI [6.22–7.22]), though there was individual variation both in average ratings (range 4.25–8.90) and in ratings for individual shocks (lowest rating overall = 2.65, highest rating overall = 10.0). In the postquestionnaire, 90% of participants answered that they could ‘never’ foresee an upcoming shock, whilst one participant answered ‘sometimes’ on the multiple‐choice question.

### 3.4. Shock Interference

Two EMA items, measured once at the end of the day, assessed how much attention was allocated towards the watch or upcoming shocks and how much the shocks interfered with activities throughout the day. Attention to the watch or upcoming shocks had a mean of 4.2 (0–10 VAS; SD = 2.59, range = 0–7.6, 95% CI [2.39–6.09]), whereas shock interference had a mean of 2.05 (0–10 VAS; SD = 1.52, range = 0–3.7, 95% CI [0.96–3.14]), indicating overall low interference of the stimulus on daily activities, even for participants who paid more attention to the watch during the day. At the last study visit, participants reported retrospectively an average shock interference of 2.3 (0–10 VAS; SD = 2.00, range = 0.0–5.9, 95% CI [0.83–3.69]), consistent with their EMA ratings. Two participants (20%) reported retrospectively (poststudy) that they avoided some activities or plans because of the shocks, whilst the remaining 8 participants (80%) avoided none.

### 3.5. Debrief Interviews

At the final study visit, participants completed short debrief interviews where they were asked a series of questions about what it was like to take part. Responses are summarised below according to each question’s theme.

#### 3.5.1. General Experience of Wearing the Watch

All participants described the experience of wearing the watch for one day as generally unproblematic. Four participants expressed that shocks were somewhat annoying and distracting at times—especially towards the end of the day. However, others reported forgetting about the shocks throughout the day and feeling very surprised when a shock occurred. One participant said they sometimes felt like they had received shocks outside of the scheduled times, whilst another reported seeing the questionnaire notification without having felt a shock. It was also mentioned by one participant that the shock intensity felt different throughout the day.

#### 3.5.2. Quality of Pain Sensation

Most participants (*N* = 6) compared the sensation of the shocks to something familiar (i.e., resembling a wasp sting, joint pain, muscle constriction, pinch, touching an electric fence or static electricity). A few participants (*N* = 4) described the sensation as distinct and unfamiliar. One participant expressed difficulty in describing the sensation: ‘[It] was often more startling than painful, so it is difficult to describe the feeling of the shock itself’. Another participant mentioned clear differences between the shock and other types of pain: ‘[The sensation] does not feel like any other pain. If it was physical pain, then it was very sudden. Most pains come gradually, and you have an idea of when it will come ‐ [such as] with movement’. The sensation was described by another participant as ‘[The pain was] radiating up the arm and pulsating out towards the elbow’.

#### 3.5.3. Disruptiveness of Shocks

None of the participants described any significant changes to daily routines due to the shocks, other than what was required to adhere to participation requirements (e.g., not driving a car, stopping current activity to disable alarm and respond to questionnaire). Some overall interference was expressed, such as having trouble relaxing, being distracted from conversations, or being more cautious when cooking. Three participants reported that they would have driven a vehicle if they had not been participating in the study that day, with one saying: ‘[It] would have been impractical if I had meetings or work’.

#### 3.5.4. Predictability of Shocks

Overall, participants reported being unable to predict shock onsets throughout the day. Yet, some expressed general expectancies related to knowing they would receive shocks during the day (i.e., ‘[I] expected it to come. [I] thought it was likely, but I did not know’; ‘I knew that I would get shocked’; ‘[The shocks were] a bit surprising, [but I] knew that they would come’; ‘[I] wondered whether something was wrong in the beginning as I expected [the shocks] to start sooner’). One participant reported incidentally predicting one shock ‘One time I randomly thought it had been a while [since the last shock]. I looked at the phone, and [the shock] suddenly came’.

#### 3.5.5. Reasons for Removing Watch

Half of the participants (*N* = 5) reported removing the watch briefly at some point during the day of participation. Reasons for removing the watch included showering, exercising using heavy weights, and baking. None of the participants removed the watch because of shock unpleasantness.

#### 3.5.6. Answering EMA Questionnaires

Most participants expressed no issue with responding to the EMA questionnaires, and the questionnaires were described as short and simple. One participant reported a positive experience of an increased awareness of their mood throughout the day from answering the questionnaires. Other participants mentioned minor issues, such as delays between when the shock was felt and when the questionnaire was available (up to 1 min), which was experienced as slightly disruptive. Moreover, one participant found it difficult to rate their emotional state whilst another mentioned that the questionnaires were a bit much after having to fill them in repeatedly throughout the day.

#### 3.5.7. Willingness to Wear the Watch Again

All participants answered that they would hypothetically be willing to wear the watch over a period of 3 days, though two expressed hesitations, saying ‘[It] depends on how strong the shocks are’ or that they ‘Probably would have [worn it for 3 days], but it would be annoying’.

### 3.6. Exploratory Analysis

#### 3.6.1. Mood Ratings

**FIGURE 1:**
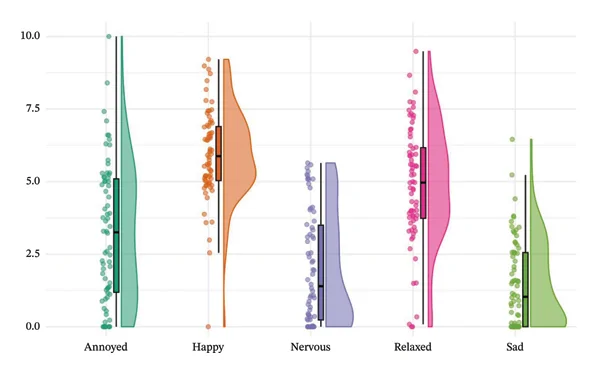
Raincloud plots of the distribution of responses for mood items for all participants.

Five different mood items were assessed in the EMA questionnaire following each shock. To investigate possible ceiling and flooring effects, the response distributions for each item are plotted visually in Figure 1. The items ‘Annoyed’(*M* = 3.23, SD = 2.40, range = 0–10.0, 95% CI [2.66–3.80]), ‘Happy’ (*M* = 5.92, SD = 1.56, range = 0–9.21, 95% CI [5.54–6.29]) and ‘Relaxed’ (*M* = 4.87, SD = 2.05, range = 0–9.49, 95% CI [4.38–5.36]) showed the widest spread of responses, whereas the items ‘Nervous’ (*M* = 2.02, SD = 1.94, range = 0–5.64, 95% CI [1.55–2.48]) and ‘Sad’ (*M* = 1.43, SD = 1.52, range = 0–6.46, 95% CI [1.07–1.79]) showed tendencies towards flooring effects.

#### 3.6.2. Time‐of‐Day Effects

**FIGURE 2:**
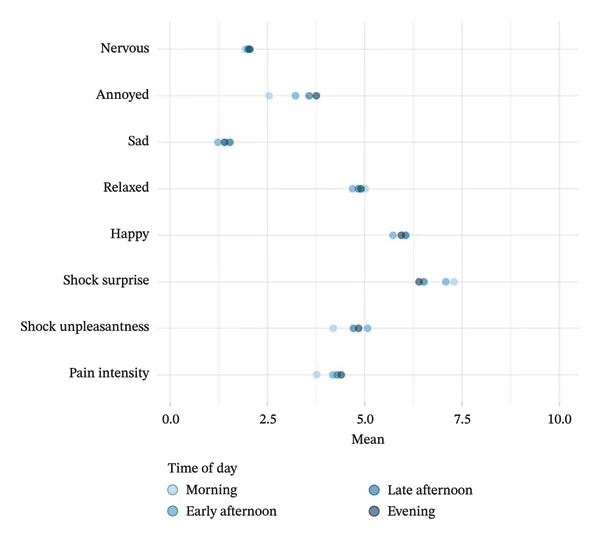
Average scores for mood and stimulus‐related variables across participants, binned into four different timepoints based on time of day. All variables were recorded on a digital VAS 0–10 scale.

To explore potential differences in time of day on EMA ratings, EMA questionnaires were binned into four different time periods based on what time of day the questionnaire was sent. A total of 14 unique timepoints (combination of Protocols A and B) were divided into the following bins: Morning (10:00–12:59), Early Afternoon (13:00–15:59), Late Afternoon (16–18:29) and Evening (18:30–21). The total number of EMA questionnaires in each time bin was *N* = 15 for morning and late afternoon, and *N* = 20 for early afternoon and evening, with *N* = 70 overall. Individual ratings for each EMA item were first averaged for each of the four time bins, before averaging across individuals for group‐level analysis. A visual presentation of group averages for all repeated EMA variables at different times of day is shown in Figure 2.

Based on the pooled standard deviation across participants, ‘Annoyed’ (SD_Pooled_ = 1.91; range 0.42–4.38, 95% CI [0.68–2.39]) and ‘Relaxed’ (SD_Pooled_ = 1.74; range 0.39–2.76, 95% CI [1.03–2.12]) showed the highest variability, followed by ‘Shock Unpleasantness’ (SD_Pooled_ = 1.49; range 0.49–2.38, 95% CI [0.93–1.81]), ‘Shock Surprise’ (SD_Pooled_ = 1.45; range 0.59–2.08, 95% CI [1.04–1.72]), ‘Pain Intensity’ (SD_Pooled_ = 1.37; range 0.36–2.28, 95% CI [0.68–1.70]) and ‘Happy’ (SD_Pooled_ = 1.08; range 0.39–1.99, 95% CI [0.67–1.32]), whereas ‘Nervous’ (SD_Pooled_ = 0.87; range 0.14–1.66, 95% CI [0.40–1.08]) and ‘Sad’ (SD_Pooled_ = 0.84; range 0–1.35, 95% CI [0.31–1.05]) showed the lowest variability.

#### 3.6.3. Exploratory Correlations

To explore potential associations between reported mood and stimulus‐related variables, within‐individual correlations were performed and summarised on the group level by calculating the mean correlation coefficient from Fisher z‐transformed individual coefficients and back‐translating into Pearson’s rho. Correlation coefficients were descriptive and hypothesis‐generating, and therefore not evaluated for statistical significance (i.e., no *p*‐values were calculated). On average, there was a strong positive correlation between shock unpleasantness and reported pain intensity, *r*_*m*_ = 0.86, range = 0.20–0.99, 95% CI [0.61–0.94], and only one participant had a correlation coefficient below *r*_*m*_ = 0.60. Shock surprise showed on average a moderate positive association with pain intensity, *r*_*m*_ = 0.40, although there was considerable individual variation (range = −0.26–0.85, 95% CI [0.0004–0.63]). An overview of the average correlation coefficients can be seen in Table 3.

**TABLE 3:** Average correlation coefficients across individuals were calculated by z‐transforming individual Pearson correlation coefficients and taking the group mean before back‐translating the Fisher z‐transformed coefficient to Pearson’s rho.

	Pain intensity	Shock unpleasantness	Shock surprise
Shock unpleasantness	0.86		
Shock surprise	0.40	0.58	
Happy	−0.09	−0.01	0.17
Relaxed	−0.16	−0.30	−0.06
Sad	−0.17^a^	0.01^a^	0.004^a^
Annoyed	0.42	0.56	0.12
Nervous	0.43	0.34	0.23

^a^For correlations with ‘sad’, one participant had a standard deviation of 0 (i.e., all responses rated 0), so a correlation coefficient was not available. This individual was, therefore, excluded from the group mean calculation, leaving a sample of *N* = 9 for correlations involving sadness.

## 4. Discussion

This pilot study investigated the tolerability, feasibility and construct validity of a novel method of administering experimental evoked pain to pain‐free individuals. Overall, participants showed high tolerability, good adherence to the study protocol, and no participants withdrew or reported any adverse events during participation. Both preregistered criteria for feasibility were met, along with the third criterion relating to construct validity, where a minimum of 75% of shocks should be rated above 1.5 on a 0–10 VAS scale as assessed by pooled ratings across participants (84.1% of shocks > 1.5). When taking the average of individual percentages of shocks rated above 1.5 (Table 2), which could have been an alternative approach, the criterion was still met (82.8% of shocks > 1.5), supporting the conclusion that the electrical stimulation sufficiently and reliably invokes pain in an ecologically valid context. Together, these results support the safety, feasibility and validity of our method of remote pain administration for future studies in healthy volunteers.

### 4.1. Interference

Overall, participants reported the shocks had relatively low interference on daily activities, in EMA‐based end‐of‐day ratings and retrospectively (poststudy), and most stated they completed plans as normal. This may be due to the brief duration of the shock stimuli (∼1 s) and participation overall (1 day). However, since both measures of interference were only rated retrospectively, there is a risk of recall bias, and conclusions about the disruptiveness of shocks may be over‐ or underestimated. It is also important to note that reported interference may not take into account activities that were restricted during the study (i.e., driving a car, using machines) as participants may already have planned to avoid these. In fact, a few participants mentioned that not being able to drive a car might pose limitations for longer participation. This is therefore a key limitation of this method if applied over several days.

### 4.2. Pain Intensity and Shock Unpleasantness

The pain intensity of the electric shocks was on average rated 4.1 (VAS: 0–10), which was very close to the target calibrated pain intensity of 4.0 (verbal NRS: 0–10), indicating that the calibration procedure was largely successful on the group level. However, four participants reported that the shock stimuli in general were a bit too mild, and three of these had an average pain intensity below 3.0. Though most participants felt all shocks, three participants missed some shocks throughout the day. It is possible that these were undetected because the shocks were too mild, which would imply the calibration procedure may not have been optimal for all participants. Another putative explanation is that the different contexts of the calibration procedure relative to at‐home participation may have influenced pain intensity ratings, e.g., through attention processes.

On the group level, pain intensity and shock unpleasantness showed a strong positive correlation, although there was substantial individual variation. This indicates that for some participants, shocks were relatively unpleasant but less painful, or vice versa. Some participants mentioned similar experiences during the debrief, saying shocks were ‘surprising’ or ‘annoying’ rather than particularly painful, whereas for other participants, the two variables were rated nearly identical.

### 4.3. Validity of the Shock Stimuli

Shocks were described by most participants as qualitatively different from most of the naturally occurring pain they were used to, yet some described similarities with past pain experiences (e.g., joint pain, bee sting, getting pinched). Clinically, neuropathic pain has been described as resembling the sensation of electric shocks [38, 39], which could indicate that our model has the potential to capture some features of this type of clinical pain. Since we enrolled pain‐free individuals in this study, the sensation of neuropathic pain may not have been recognisable and therefore not mentioned when asked about similar pain sensations. However, one participant did describe the pain as radiating and pulsating, which resembles clinical descriptions of nerve pain [40]. In general, when the goal is to gain knowledge relevant to clinical pain, an experimental model that evokes sensations *resembling clinical pain* would increase face validity. This could be done by matching aspects such as location, duration and the quality of the pain sensation (i.e., stinging, throbbing).

One notable anecdote from a participant was that they sometimes felt like they had been shocked in the absence of an actual shock stimulus, consistent with an expectation‐ or attention‐induced (mild) hallucination akin to a phantom sensation. In future, this model may be used to investigate the mechanisms of phantom sensations or placebo/nocebo effects, for example, by using predictive cues in a classical conditioning paradigm to see how cues alone may produce stimulus‐independent somatic sensations.

### 4.4. Within‐Day Variability

One participant noted that they felt like shocks were different in strength throughout the day, which points to a difference in stimulus perception throughout the day. On the group level, aggregated average ratings showed slightly lower pain intensity and shock unpleasantness ratings for shocks given in the morning compared to later in the day. Further research, e.g., using a multiday paradigm, is needed to evaluate whether this is due to morning‐specific factors or whether this is because of stimulus order. Additionally, average shock surprise showed a decreasing trend throughout the day, possibly reflecting how participants became more familiar with the stimulus from repeated exposure. Though exploratory, these results highlight the importance of further research into time‐of‐day effects on stimulus perception, using wearable devices for evoked pain where longer testing durations are possible.

### 4.5. Limitations

Our study has several limitations. First, the sample was limited both in size and in generalisability to the wider population in terms of age and other demographic factors. Second, the watch used for electrical stimulation has inherent limitations as a wearable device for experimentally evoked pain. For one, each electrical stimulus can only be set to a single intensity, precluding the use of stimuli with ramping or fluctuating intensity. The stimulus is also restricted to the wrist only, as the device did not allow for flexible placement; however, this may be solved with customised straps or other attachments. Third, participants had to manually disable shocks using a button press to avoid additional shocks. The exact number of missed initial shocks could therefore be inaccurate, as some shocks may have been detected during repetitions but reported as first‐time shocks.

Lastly, there are important differences between any experimental model and clinical pain. Experimental pain, even when administered in day‐to‐day environments, will naturally hold lower stakes due to reduced uncertainty around the cause, duration, and meaning of pain [23]. Our model attempts to address these limitations by having participants perform daily tasks during evoked pain and extending the window of possible stimulus onset to increase uncertainty and reduce predictability of the pain stimulus. However, the cause of pain in our model is still a known safe external source (i.e., electric shocks from the watch) and the watch can be easily removed and is worn only for a brief time. Together, these elements lower the perceived threat value, compared to unknown pains that originate within the body. It is therefore important not to generalise findings using this method to clinical populations but rather apply the model as an additional tool for investigating basic pain mechanisms.

### 4.6. Future Studies

To better understand the potential and limitations of this model of remote evoked pain, future studies should aim to test pain administration over longer periods of time, in a larger and more diverse sample, and investigate associations with variables that may impact pain perception. For example, it might be beneficial to look at the effect of different types of daily activities (i.e., exercise, work, passive leisure) on pain by asking participants to report what they were doing when they received a shock. Previous research indicates that factors such as social contact [41] and physical activity [42, 43] are relevant in clinical fluctuations in pain. Further research is needed to test whether these influences translate to experimental pain. Repeated measurements of shock interference would also support construct validity in future work, by capturing possible fluctuations in disruptiveness of shocks depending on context or time of day.

Future studies could also look at the effect of manipulating features of the pain stimulus, such as different shock intensities, repeated shocks, and onset predictability. Over time, it would also be beneficial to develop a specialised wearable device that is designed for experimental evoked pain with features such as gradual stimulus onset and automatic termination, preprogrammed stimulus patterns (e.g., mimicking naturalistic ‘waves’ of pain), and more flexible device placement. This would allow for better manipulation of stimulus variables and would allow for the exploration of research questions specific to different types of pain—such as lower back pain or pelvic pain.

## 5. Conclusion

This pilot feasibility study investigated a novel model of experimental evoked pain in a small sample of pain‐free individuals for the duration of one day. Our results suggest that this method of administering experimental pain in a more ecologically valid setting is feasible and tolerable for participants for a short duration. More research is needed to establish the usefulness and applicability of this model in investigating key parameters of the pain experience such as stimulus predictability, mood influences, and temporal variability, especially over longer periods of time.

## Funding

This study was funded by the University of Oslo Growth House (2023/12281), South‐Eastern Norway Regional Health Authority (2020040 and 2022086) and Foundation Dam (SDAM_FOR555652).

## Conflicts of Interest

The authors declare no conflicts of interest.

## Supporting Information

Additional supporting information can be found online in the Supporting Information section.

## Supporting information


**Supporting Information** Further description of Time Protocols A and B and results of average pain intensity and shock unpleasantness for each protocol. The file includes Supporting Table 1, which outlines the timepoints of scheduled shocks for each protocol, and Supporting Figure 1, which visualises differences in ratings of pain intensity, shock surprise and shock unpleasantness for each protocol.

## Data Availability

The data that support the findings of this study are available from the corresponding author upon request.

## References

[1] Gong W. , Fan L. , and Luo F. , Does Experimentally Induced Pain Affect Attention? A meta-analytical Review, J Pain Res Dove Medical Press. (2019) 12, 585–595, 10.2147/jpr.s184183. PMC6368116 30787635

[2] Kyle B. N. and McNeil D. W. , Autonomic Arousal and Experimentally Induced Pain: a Critical Review of the Literature, Pain Research and Management. (2014) 19, no. 3, 536859–167, 10.1155/2014/536859. PMC4158962 24533429

[3] Mischkowski D. , Stavish C. M. , Palacios-Barrios E. E. , Banker L. A. , Dildine T. C. , and Atlas L. Y. , Dispositional Mindfulness and Acute Heat Pain: Comparing Stimulus-Evoked Pain with Summary Pain Assessment, Biopsychosoc Sci Med. (2021) 83, no. 6, 539–548, 10.1097/psy.0000000000000911. PMC8805938 34213859

[4] Ellingsen D.-M. , Wessberg J. , Eikemo M. et al., Placebo Improves Pleasure and Pain Through Opposite Modulation of Sensory Processing, Proceedings of the National Academy of Sciences of the United States of America. (2013) 110, no. 44, 17993–17998, 10.1073/pnas.1305050110. PMC3816412 24127578

[5] Nir R.-R. , Granovsky Y. , Yarnitsky D. , Sprecher E. , and Granot M. , A Psychophysical Study of Endogenous Analgesia: the Role of the Conditioning Pain in the Induction and Magnitude of Conditioned Pain Modulation, European Journal of Pain. (2011) 15, no. 5, 491–497, 10.1016/j.ejpain.2010.10.001. 21035364

[6] Rainville P. , Bao Q. V. H. , and Chrétien P. , Pain-Related Emotions Modulate Experimental Pain Perception and Autonomic Responses, Pain. (2005) 118, no. 3, 306–318, 10.1016/j.pain.2005.08.022. 16289802

[7] Werner M. U. , Pereira M. P. , Andersen L. P. H. , and Dahl J. B. , Endogenous Opioid Antagonism in Physiological Experimental Pain Models: a Systematic Review, PLOS ONE Public Library of Science. (2015) 10, no. 6, 10.1371/journal.pone.0125887. PMC4452333 26029906

[8] Anker-møller E. , Spangsberg N. , Arendt-nielsen L. , Schultz P. , Kristensen M. S. , and Bjerring P. , SUBHYPNOTIC DOSES OF THIOPENTONE AND PROPOFOL CAUSE ANALGESIA TO EXPERIMENTALLY INDUCED ACUTE PAIN, British Journal of Anaesthesia. (1991) 66, no. 2, 185–188, 10.1093/bja/66.2.185. 1817618

[9] Ellingsen D.-M. , Isenburg K. , Jung C. et al., Dynamic brain-to-brain Concordance and Behavioral Mirroring as a Mechanism of the patient-clinician Interaction, Science Advances. (2020) 6, no. 43, 10.1126/sciadv.abc1304. PMC7577722 33087365

[10] Price D. D. , Milling L. S. , Kirsch I. , Duff A. , Montgomery G. H. , and Nicholls S. S. , An Analysis of Factors that Contribute to the Magnitude of Placebo Analgesia in an Experimental Paradigm, Pain. (1999) 83, no. 2, 147–156, 10.1016/s0304-3959(99)00081-0. 10534585

[11] Staahl C. , Olesen A. E. , Andresen T. , Arendt-Nielsen L. , and Drewes A. M. , Assessing Analgesic Actions of Opioids by Experimental Pain Models in Healthy Volunteers–an Updated Review, British Journal of Clinical Pharmacology. (2009) 68, no. 2, 149–168, 10.1111/j.1365-2125.2009.03456.x. PMC2767277 19694733

[12] Szikszay T. M. , Adamczyk W. M. , Hoegner A. , Woermann N. , and Luedtke K. , The Effect of acute-experimental Pain Models on Offset Analgesia, European Journal of Pain. (2021) 25, no. 5, 1150–1161, 10.1002/ejp.1740. 33533139

[13] Edwards R. R. , Sarlani E. , Wesselmann U. , and Fillingim R. B. , Quantitative Assessment of Experimental Pain Perception: Multiple Domains of Clinical Relevance, Pain. (2005) 114, no. 3, 315–319, 10.1016/j.pain.2005.01.007. 15777856

[14] Bulls H. W. , Freeman E. L. , Anderson A. J. , Robbins M. T. , Ness T. J. , and Goodin B. R. , Sex Differences in Experimental Measures of Pain Sensitivity and Endogenous Pain Inhibition, J Pain Res Dove Medical Press. (2015) 8, 311–320, 10.2147/jpr.s84607. PMC4494610 26170713

[15] Fillingim R. B. , Sex Differences in Analgesic Responses: Evidence from Experimental Pain Models, Eur J Anaesthesiol EJA. (2002) 19, no. Supplement 26, 16–24, 10.1097/00003643-200219261-00004. 12512212

[16] Vogel K. , Muhammad L. N. , Song J. et al., Sex Differences in Pain and Quantitative Sensory Testing in Patients with Rheumatoid Arthritis, Arthritis Care & Research. (2023) 75, no. 12, 2472–2480, 10.1002/acr.25178. PMC10704379 37365745

[17] Cheng J. C. , Anzolin A. , Berry M. et al., Dynamic Functional Brain Connectivity Underlying Temporal Summation of Pain in Fibromyalgia, Arthritis & Rheumatology. (2022) 74, no. 4, 700–710, 10.1002/art.42013. 34725971

[18] Tham S. W. , Palermo T. M. , Holley A. L. et al., A Population-based Study of Quantitative Sensory Testing in Adolescents with and Without Chronic Pain, Pain. (2016) 157, no. 12, 2807–2815, 10.1097/j.pain.0000000000000716. PMC13585038 27780176

[19] Uddin Z. and MacDermid J. C. , Quantitative Sensory Testing in Chronic Musculoskeletal Pain, Pain Medicine. (2016) 17, no. 9, 1694–1703, 10.1093/pm/pnv105. 26893116

[20] Weaver K. R. , Griffioen M. A. , Klinedinst N. J. et al., Quantitative Sensory Testing Across Chronic Pain Conditions and Use in Special Populations, Front Pain Res [Internet] Frontiers. (2022) 2, 10.3389/fpain.2021.779068. PMC8915716 35295425

[21] Rabey M. , Kendell M. , Koren S. et al., Do Chronic Low Back Pain Subgroups Derived from Dynamic Quantitative Sensory Testing Exhibit Differing Multidimensional Profiles?, Scand J Pain De Gruyter. (2021) 21, no. 3, 474–484, 10.1515/sjpain-2020-0126. 33639047

[22] Reimer M. , Forstenpointner J. , Hartmann A. et al., Sensory Bedside Testing: a Simple Stratification Approach for Sensory Phenotyping, PAIN Rep. (2020) 5, no. 3, 10.1097/pr9.0000000000000820. PMC7447375 32903958

[23] Edens J. L. and Gil K. M. , Experimental Induction of Pain: Utility in the Study of Clinical Pain, Behavior Therapy. (1995) 26, no. 2, 197–216, 10.1016/s0005-7894(05)80102-9.

[24] May M. , Junghaenel D. U. , Ono M. , Stone A. A. , and Schneider S. , Ecological Momentary Assessment Methodology in Chronic Pain Research: a Systematic Review, Journal of Pain. (2018) 19, no. 7, 699–716, 10.1016/j.jpain.2018.01.006. PMC6026050 29371113

[25] Rogers A. H. , Smit T. , Bakhshaie J. , and Zvolensky M. J. , Impact of Intraindividual Pain Variability on Functional Pain Outcomes Among Adults with Chronic Pain: an Ecological Momentary Assessment Study, Journal of Behavioral Medicine. (2025) 48, no. 6, 1035–1042, 10.1007/s10865-025-00590-x. PMC12589202 40779061

[26] Icmje , Recommendations | Clinical Trials [Internet], International Committee of Medical Journal. https://www.icmje.org/recommendations/browse/publishing-and-editorial-issues/clinical-trial-registration.html.

[27] Behavioral Technology Group , Pavlok 3 Pro, Pavlok Store. (2026) https://shop.pavlok.com/products/pavlok3.

[28] Mestdagh M. , Verdonck S. , Piot M. et al., m-Path: an easy-to-use and Highly Tailorable Platform for Ecological Momentary Assessment and Intervention in Behavioral Research and Clinical Practice, Frontiers in Digital Health. (2023) 5, 10.3389/fdgth.2023.1182175. PMC10619650 37920867

[29] m-Path Software , m-Path, 2025, https://www.m-Path.io.

[30] Sullivan M. J. L. , Bishop S. R. , and Pivik J. , The Pain Catastrophizing Scale: Development and Validation, Psychol Assess American Psychological Association. (1995) 7, no. 4, 524–532, 10.1037/1040-3590.7.4.524.

[31] Spitzer R. L. , Kroenke K. , Williams J. B. W. , and Löwe B. , A Brief Measure for Assessing Generalized Anxiety Disorder: the GAD-7, Archives of Internal Medicine. (2006) 166, no. 10, 1092–1097, 10.1001/archinte.166.10.1092. 16717171

[32] Beck A. T. , Steer R. A. , and Brown G. , Beck Depression Inventory–II, Psychological Assessment. (1996) https://doi.apa.org/doi/10.1037/t00742-000.

[33] Scheier M. F. and Carver C. S. , Bridges MW: Distinguishing Optimism from Neuroticism (And Trait Anxiety, self-mastery, and self-esteem): a Reevaluation of the Life Orientation Test, J Pers Soc Psychol US: American Psychological Association. (1994) 67, no. 6, 1063–1078, 10.1037//0022-3514.67.6.1063. 7815302

[34] Cohen S. , Kamarck T. , and Mermelstein R. , A Global Measure of Perceived Stress, J Health Soc Behav JSTOR. (1983) 24, no. 4, 385–396, 10.2307/2136404. 6668417

[35] Fugl-Meyer A. R. , Melin R. , and Fugl-Meyer K. S. , Life Satisfaction in 18-to 64-year-old Swedes: in Relation to Gender, Age, Partner and Immigrant Status, Journal of Rehabilitation Medicine. (2002) 34, no. 5, 239–246, 10.1080/165019702760279242. 12392240

[36] R Core Team , R: A Language and Environment for Statistical Computing [Internet], 2022, R Foundation for Statistical Computing, Vienna, Austria, https://www.R-project.org/.

[37] Wickham H. , ggplot2: Elegant Graphics for Data Analysis, 2016, Springer-Verlag, New York, https://ggplot2.tidyverse.org.

[38] Baron R. , Canning B. J. and Spina D. , Neuropathic Pain: a Clinical Perspective, Sens Nerves [Internet], Springer, Berlin, Heidelberg, 3–302009, 10.1007/978-3-540-79090-7_1.

[39] Truini A. , A Review of Neuropathic Pain: from Diagnostic Tests to Mechanisms, Pain and Therapy. (2017) 6, no. S1, 5–9, 10.1007/s40122-017-0085-2. PMC5701892 29178037

[40] Neal S. L. and Fields K. B. , Peripheral Nerve Entrapment and Injury in the Upper Extremity, American Family Physician. (2010) 81, 147–155. 20082510

[41] Weiß M. , Gründahl M. , Jachnik A. et al., The Effect of Everyday-Life Social Contact on Pain, 2024, Gunther Eysenbach MD MPH, Associate Professor. 10.2196/53830 PMC11094601 38687594

[42] Chang A. H. , Hertel E. , Bruun M. K. , Kristensen E. M. , Petersen K. K.-S. , and Rathleff M. S. , Temporal Associations of Physical Activity with Subsequent Knee Pain in Individuals with Knee Osteoarthritis: an Ecological Momentary Assessment Study, European Journal of Pain. (2025) 29, no. 6, 10.1002/ejp.70026. PMC12032517 40285396

[43] Tynan M. , Virzi N. , Wooldridge J. S. , Morse J. L. , and Herbert M. S. , Examining the Association Between Objective Physical Activity and Momentary Pain: a Systematic Review of Studies Using Ambulatory Assessment, Journal of Pain. (2024) 25, no. 4, 862–874, 10.1016/j.jpain.2023.10.021. 37914094

