# Supplementary material for: An Ecologically Valid Experimental Model for Repeated Evoked Pain Using a Wearable Device in Pain‐Free Individuals: A Pilot Feasibility Study
Source: Pain Res Manag. 2026 Sep 22;2026:9255164. doi: 10.1155/prm/9255164 (PMC13598433; doi:10.1155/prm/9255164)
Supplement: Supplementary file 1 [file PRM-2026-9255164-s001.pdf]

Supplementary Info:

1.0 Time protocols (A and B)

Participants were randomised (1:1) to one of the following protocols to vary the exact timepoints of each shock. Timepoints were created in a pseudorandomized fashion, using a random timepoint generator, with the restriction that there should be at least 30 minutes between each shock (see Supplementary Table 1). Visual inspection of differences between protocols on stimulus-related variables showed a possible trend of higher participant ratings in Protocol B for pain intensity ($M_{Protocol\ B}$ = 4.85, $SD_{Protocol\ B}$ = 1.55; $M_{Protocol\ A}$ = 3.35, $SD_{Protocol\ A}$ = 1.48) and shock unpleasantness ($M_{Protocol\ B}$ = 5.27, $SD_{Protocol\ B}$ = 1.77; $M_{Protocol\ A}$ = 3.92, $SD_{Protocol\ A}$ = 1.42), compared to Protocol A (see Supplementary Figure 1). Due to the small sample size in each group (*n* = 5) it is not possible to conclude whether this was due to the different time-protocols. Thus, data from both protocols were analysed together.

Shock Number	Protocol A	Protocol B
1	10:49	11:11
2	11:34	13:02
3	13:53	15:50
4	15:49	17:08
5	17:04	18:01
6	18:35	18:33
7	20:27	19:27

Supplementary Table 1: Overview of timepoints when a shock was delivered for each protocol.

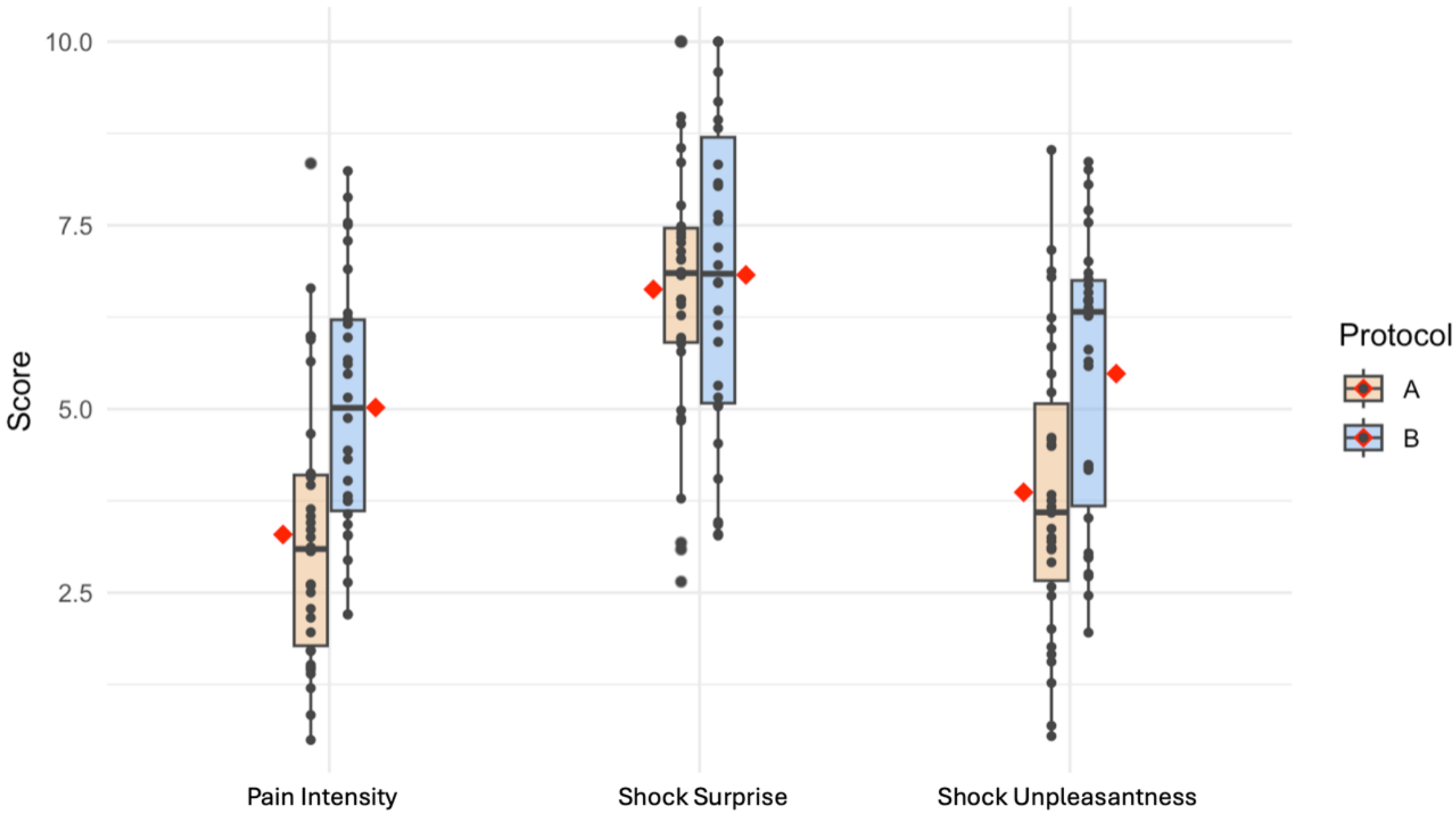

Supplementary Figure 1: Differences in rating on stimulus-related variables for protocol A and B for all shocks. Red shapes indicate group mean.
